# Pre-treatment neutrophil-to-lymphocyte ratio is associated with neutrophil and T-cell infiltration and predicts clinical outcome in patients with glioblastoma

**DOI:** 10.1186/s12885-015-1629-7

**Published:** 2015-09-04

**Authors:** Sheng Han, Yang Liu, Qingchang Li, Zhonghua Li, Haipei Hou, Anhua Wu

**Affiliations:** 1 Department of Neurosurgery, The First Hospital of China Medical University, Nanjing Street 155, Heping District, Shenyang, 110001 China; 2 Department of Pathology, China Medical University, Shenyang, 110001 China

## Abstract

**Background:**

Markers of systemic inflammation are correlated with patient survival in various cancers. The prognostic value of neutrophil-to-lymphocyte ratio (NLR) was compared with that of platelet-to-lymphocyte ratio (PLR) in patients with glioblastoma. The association of NLR with neutrophil and T- cell infiltration was also explored.

**Methods:**

A total of 152 patients with glioblastoma were retrospectively analyzed. Clinical information was obtained from electronic medical records. Kaplan-Meier analysis and the Cox proportional hazards models were used to examine the survival function of pre-treatment NLR and PLR in these glioblastoma patients. Neutrophil and CD3^+^ T-cell infiltration was assessed by immunohistochemical staining of tissue microarray cores from glioblastomas.

**Results:**

Pre-treatment NLR levels were significantly correlated with overall survival (OS) in glioblastoma patients (multivariate hazard ratio =1.050; 95 % confidence interval, 1.003–1.100; *P* = 0.037). Despite the correlation between NLR and PLR (*R* = 0.509, *P* < 0.001), NLR was superior to PLR as a prognostic factor. High pre-treatment NLR (≥4 versus < 4) was significantly associated with high neutrophil infiltration and low CD3^+^ T-cell infiltration into tumors, and predicted poor OS (mean, 10.6 *vs.* 17.9 months, *P* < 0.001).

**Conclusions:**

Pre-treatment NLR is of prognostic significance independent of MGMT status and is superior to PLR as a prognostic factor. Our results demonstrate a correlation between elevated peripheral blood NLR levels and increased tumor neutrophil infiltration/decreased CD3^+^ T-cell infiltration.

## Background

Glioblastoma is the most common malignant primary brain tumor in adults [1]. Currently, the standard treatment for glioblastoma patients is debulking surgery combined with radiotherapy and temozolomide (TMZ) chemotherapy [2]. Despite receiving the same treatment, glioblastoma patients show significant variation in their clinical outcomes because of the heterogeneity of the tumors and multiple systemic factors [3]. Therefore, prognostic markers that can guide individual adjuvant therapy and follow-up schedule are urgently needed. Prognostic markers in the peripheral blood are of considerable clinical value because of their accessibility [4].

Tumor-infiltrating immune cells, including neutrophils and lymphocytes, play an important role in glioblastoma progression and prognosis [5–10], reflecting the significance of local inflammatory factors. However, the heterogeneity in the amounts and the spatial localization of tumor-infiltrating immune cells, both between and within the patients’ tumors, limits their clinical use as prognostic markers [7, 11]. Recently, the prognostic value of neutrophil-to-lymphocyte ratio (NLR) in peripheral blood, a marker of systemic inflammatory responses, was identified in glioblastoma [12, 13] and in many other types of cancer [14–16]. In patients with glioblastoma, NLR >4 is an independent prognostic indicator of poor outcome [12, 13]. These results raise the question of whether another inflammatory marker, the platelet-to-lymphocyte ratio (PLR) [15, 17], also has prognostic value in glioblastomas. Therefore, the influence of dynamic changes on the prognostic significance of NLR should be further examined, and the correlation between local and systemic inflammatory markers should be explored. In addition, the potential effects of the molecular marker *O*-methylguanine-DNA methyltransferase (MGMT) on the prognostic role of NLR remain unclear. In the present study, we retrospectively analyzed 152 patients with glioblastoma treated in our neurosurgical center to evaluate the prognostic value of NLR and PLR. The association of NLR with neutrophil and T-cell infiltration was also assessed.

## Methods

### Study population

Clinical samples and patient records corresponding to 217 consecutive patients diagnosed with glioblastoma at the Neurosurgery Department of the First Hospital of China Medical University between January 2010 and October 2014 were examined. Patients with other diseases, including diabetes mellitus, metabolic syndrome [18, 19], heart disease (acute coronary syndromes, rheumatic or congenital heart disease and cardiomyopathy), hypertension [20–22], severe renal or hepatic dysfunction, other cancers, inflammatory diseases, previous history of infection within 3 months and any medication usage related to inflammatory conditions that could significantly influence NLR or survival or those lacking complete data were excluded. Finally, 152 newly diagnosed patients were included in the analysis. Patients underwent surgical resection by neurosurgeons, who used similar operational techniques and principles. Tumor samples were immediately snap-frozen in liquid nitrogen after resection. One part of each sample was fixed with formalin, embedded with paraffin wax and, kept at room temperature. Glioblastomas were diagnosed by two neuropathologists according to the World Health Organization 2007 criteria. All patients received postoperative radio-chemotherapy according to the Stupp protocol [2].

Overall survival (OS) was defined as the interval between surgery and death from glioblastoma. The median follow-up period was 13 months (range, 1–53 months), during which 123 (80.9 %) patients died from glioblastoma. Data were censored at the last follow-up for patients who were alive at the time of the analysis. The present study was approved by the institutional review board of The First Hospital of China Medical University, and written informed consent was obtained from all glioma tissue donors who consented to the use of the tumor tissue and clinical data for future research. The research was in compliance with the Helsinki Declaration.

### Blood examination

Complete blood count was obtained preoperatively before any treatment (e.g., steroids) and repeated the first day after surgery as previously described [23]. Blood samples were tested by the staff at the Department of Clinical Laboratory within 2 h of collection using a Sysmex XE-2100 complete blood count analyzer (Sysmex, Kobe, Japan). The analysis included blood neutrophil, lymphocyte and platelet counts. The normal reference range is 1.8–6.3 × 10^9^/L for neutrophils, 1.1–3.2 × 10^9^/L for lymphocytes, and 125–350 × 10^9^/L for platelets. The NLR was defined as the absolute neutrophil count divided by the absolute lymphocyte count, and the PLR was defined as the absolute platelet count divided by the absolute lymphocyte count.

### Tissue microarrays and immunohistochemistry

Tissue microarrays were constructed using 152 glioblastoma clinical samples and analyzed by immunohistochemical staining as previously described [5, 24]. Samples were collected from the most phenotypically representative tumor regions. Diluted primary antibodies against human myeloperoxidase (MPO; ab134132, 1:250; Abcam, Cambridge, UK), CD15 (ab754, 1:50; Abcam) or CD3 (ab16669, 1:100; Abcam) were used and incubated overnight at 4 °C. Samples were then incubated with the horseradish peroxidase labeled secondary antibody in the immunohistochemical kit (KIT-5930, MaxVision, Fu Zhou, China) for 30 min at room temperature. Diaminobenzidine was used for color development and hematoxylin as counterstain. Results were visualized and photographed under a light microscope (Olympus BX-51; Olympus Optical Co., Ltd., Tokyo, Japan).

Semi-quantitative evaluation was performed by examining each section using at least ten randomly selected different high-power fields (HPF). Neutrophils and T-cells were identified as cells staining positive for anti-MPO and anti-CD3 antibodies, respectively, together with their characteristic morphology. Neutrophils were also verified by anti-CD15 staining. The number of infiltrating neutrophils and T-cells was manually counted independently by two investigators (YL and ZL) and an experienced neuropathologist (QL) blinded to the clinical background of the patients. The mean number of neutrophils and T-cells per field was calculated. The following scoring system was used: 0, <10 cells per field; 1, 10–20 cells per field; 2, 20–50 cells per field; 3, 50–100 cells per field; 4, 100 or more cells per field [8]. When strong differences in scoring between observers occurred, the core was re-evaluated to reach a concordant scoring [5].

### MGMT promoter methylation status

Methylation-specific PCR (MSP) was performed as previously described [5, 25] to detect MGMT promoter methylation. Briefly, tissue samples were lysed with 490 μl lysis buffer containing 20 mM Tris-Cl (pH 8.0), 5 mM EDTA (pH 8.0), 400 mM NaCl and 1 % (w/v) SDS, and digested with 10 μl proteinase K at 10 mg/ml at 37 °C for 12 h. Genomic DNA was purified from the lysate by phenol/chloroform extraction. One microgram of DNA was denatured by NaOH and modified by sodium bisulfite. MSP was performed using primer sequences for MGMT as follows: 5′-TTT GTG TTT TGA TGT TTG TAG GTT TTT GT-3′ (forward) and 5′-AAC TCC ACA CTC TTC CAA AAA CAA AAC A-3′ (reverse) for the unmethylated reaction; and 5′-TTT CGA CGT TCG TAG GTT TTC GC-3′ (forward) and 5′-GCA CTC TTC CGA AAA CGA AAC G-3′ (reverse) for the methylated reaction. Each PCR reaction (10 μl) was loaded onto nondenaturing 6 % polyacrylamide gels, stained with ethidium bromide, and visualized under UV illumination. The PCR reaction was repeated at least three times.

### Statistical analysis

Univariate and multivariate Cox proportional hazards models were constructed. Sex, age, tumor size, pre-treatment Karnofsky performance status (KPS), degree of resection, body mass index (BMI), MGMT promoter methylation, NLR, PLR, neutrophil count, platelet count and lymphocyte count were included in the analysis. To adjust for potential confounders, age, tumor size, KPS, BMI, NLR, PLR, neutrophil count, platelet count and lymphocyte count were used as continuous variables and all of the other covariates were used as categorical variables. Tumor size was calculated based on preoperative MRI scans as follows: longest diameter × widest diameter × thickness (section thickness × the number of layers) × 1/2. MGMT promoter methylation status was dichotomized (methylation vs. unmethylation). According to previous reports [5, 26], tumor resection was defined as follows: (0) biopsy or residual tumor >30 %, (1) subtotal resection with residual tumor <30 %, and (2) gross total resection. The NLR was also analyzed as a dichotomous variable, according to previous data where a NLR ≥4 (versus NLR <4) conferred a worse prognosis [12, 13]. Kaplan-Meier survival analysis was used to determine the distribution of OS time, and the results were analyzed with the log-rank test.

Pearson correlation analysis was used to examine the correlation between clinical variables. The chi-square test and ANOVA were used to determine statistical significance. The Student’s *t*-test or Mann–Whitney *U*-test was used for variables with parametric distribution or non-parametric distribution, respectively. Statistical analyses were performed with SPSS 19.0 (SPSS Inc., Chicago, IL, USA). A two-tailed *P*-value of <0.05 was regarded as significant.

## Results

Clinicopathologic data of the 152 glioblastoma patients are summarized in Table 1; 106 (69.7 %) patients were under and 46 (30.3 %) were over 60 years of age. The KPS was 70–100 in 102 (67.1 %) patients and <70 in 50 (32.9 %) patients; 95 patients (62.5 %) were male. The mean OS was 15.6 ± 11.2 months. The corresponding 1- and 2-year survival rates were 56.6 and 22.4 %, respectively.

**Table 1:** Clinical and molecular characteristics according to pre-treatment NLR in 152 glioblastoma cases

Clinical or Molecular Feature	All Cases			Pre-treatment NLR						*P*
				<4			≥4			
	No.		%	No.		%	No.		%	
Total No. of patients	152		100	103		67.8	49		32.2	
Sex										0.823
Male	95		62.5	65		68.4	30		31.6	
Female	57		37.5	38		66.7	19		33.3	
Age, years										0.136
Mean ± SD		50.4 ± 15.4			49.2 ± 16.2			53.1 ± 13.1		
≤ 60	112		73.7	80		71.4	32		28.6	0.118
> 60	40		26.3	23		57.5	17		42.5	
Tumor size, cm^3^										0.920
Mean ± SD		60.6 ± 26.6			60.4 ± 26.1			61.0 ± 28.0		
KPS										0.212
Mean ± SD		71.4 ± 14.2			70.4 ± 15.2			73.5 ± 11.6		
≤ 70	78		51.3	54		69.2	24		30.8	0.731
80–100	74		48.7	49		66.2	25		33.8	
Resection										0.659
Biopsy	38		25.0	28		73.7	10		26.3	
Subtotal	39		25.7	26		66.7	13		33.3	
Gross total	75		49.3	49		65.3	26		34.7	
BMI, kg/m^2^										0.150
Mean ± SD		24.2 ± 3.2			23.9 ± 3.3			24.8 ± 3.1		
MGMT promoter										0.975
Methylated	53		34.9	36		67.9	17		32.1	
Unmethylated	99		65.1	67		67.7	32		32.3	
Pre-treatment PLR										*<0.001*
Mean ± SD		135.0 ± 57.1			111.4 ± 34.7			184.8 ± 62.9		
OS, months										*<0.001*
Mean ± SD		15.6 ± 11.2			17.9 ± 11.0			10.6 ± 9.8		

The differences between patients with pre-treatment NLR <4 and ≥4 were compared. *P* value was calculated using chi-square test for categorical variables and using Student’s *t*-test for continuous variables

*BMI* Body Mass Index, *KPS* Karnofsky Performance Scores, *MGMT* O(6)-methylguanine-DNA-methyltransferase, *NLR* Neutrophil to Lymphocyte Ratio, *OS* Overall Survival, *PLR* Platelet to Lymphocyte Ratio

In the present study, the mean pre-treatment neutrophil, platelet and lymphocyte counts were 5.9 ± 3.6 × 10^9^/L (range, 1.2–18.6 × 10^9^/L), 222.7 ± 61.3 × 10^9^/L (range, 113–413 × 10^9^/L) and 1.8 ± 0.7 × 10^9^/L (range, 0.7–4.6 × 10^9^/L), respectively. The mean pre-treatment NLR was 4.1 ± 3.8 (median, 2.54; range, 0.7–20.6), and the pre-treatment PLR was 135.0 ± 57.1 (median, 122.1; range, 46.6–311.5). As shown in Table 1, the pre-treatment NLR did not vary significantly with sex, age, tumor size, KPS, degree of resection, BMI and MGMT promoter methylation status.

### Pre-treatment NLR and the survival of glioblastoma patients

**Fig. 1:**
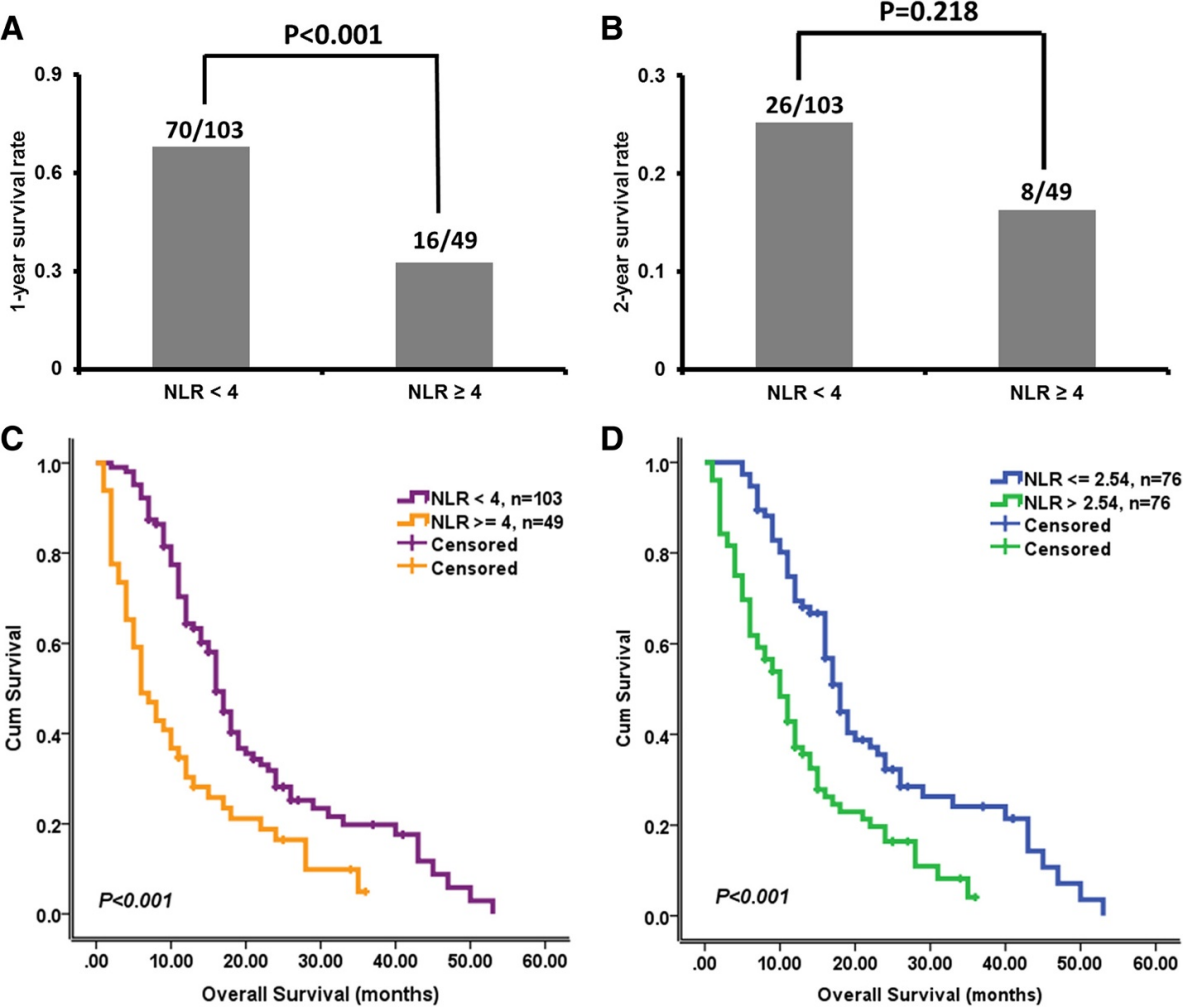
Pre-treatment neutrophil-to-lymphocyte ratio (NLR) and prognosis. **a**, **b** Pre-treatment NLR levels and 1-year (**a**) and 2-year (**b**) survival rates. **c**, **d** Kaplan-Meier survival curves stratified by pre-treatment NLR levels. Survival time was significantly shorter among patients with NLR ≥4 (or >2.54) than among those with NLR <4 (or ≤2.54)

Next, we examined the survival function of pre-treatment NLR in glioblastoma patients. Univariate and multivariate Cox regression analyses showed that pre-treatment NLR was an independent predictor of OS (multivariate hazard ratio = 1.050, 95 % confidence interval 1.003–1.100, *P* = 0.037, Table 2). As shown in Fig. 1a and b, Patients with high NLR (≥4) had lower 1-year and 2-year survival rates than those with low NLR (<4). The OS of patients with high NLR (≥4) was also shorter than that of patients with low NLR (<4; mean 10.6 *vs.* 17.9 months, *P* < 0.001; Fig. 1c). When the median pre-treatment NLR (2.54) was used as the cutoff point, similar results were obtained (Fig. 1d).

**Table 2:** Univariate and multivariate analyses of different prognostic parameters for overall survival of 152 glioblastoma patients

Variable	Univariate			Multivariate		
	*P*	HR	95 % CI	*P*	HR	95 % CI
Sex^a^	0.704	0.930	0.641–1.351	-	-	-
Age^b^	0.050	1.013	1.000–1.026	0.157	1.010	0.996–1.024
Tumor size^b^	0.559	0.998	0.991–1.005	-	-	-
KPS^b^	0.034	0.986	0.973–0.999	0.023	0.985	0.972–0.998
Resection^a^	0.047	0.800	0.641–0.997	0.183	0.860	0.688–1.074
BMI^b^	0.206	1.033	0.982–1.086	-	-	-
MGMT promoter^a^	0.011	0.605	0.411–0.890	0.041	0.659	0.442–0.984
Pretreatment PLR^b^	0.013	1.004	1.001–1.007	0.152	1.003	0.999–1.007
Postoperative NLR^b^	0.285	1.015	0.988–1.043	-	-	-
Pretreatment neutrophils^b^	0.620	1.010	0.971–1.051	-	-	-
Pretreatment lymphocytes^b^	0.531	0.999	0.996–1.002	-	-	-
Pretreatment platelets^b^	0.637	0.999	0.997–1.002	-	-	-
Pretreatment NLR^b^	<0.001	1.078	1.038–1.119	0.037	1.050	1.003–1.100

*BMI* Body Mass Index, *KPS* Karnofsky Performance Scores, *MGMT* O(6)-methylguanine-DNA-methyltransferase, *NLR* Neutrophil to Lymphocyte Ratio, *PLR* Platelet to Lymphocyte Ratio

^a^categorical variable; ^b^continuous variable

**Fig. 2:**
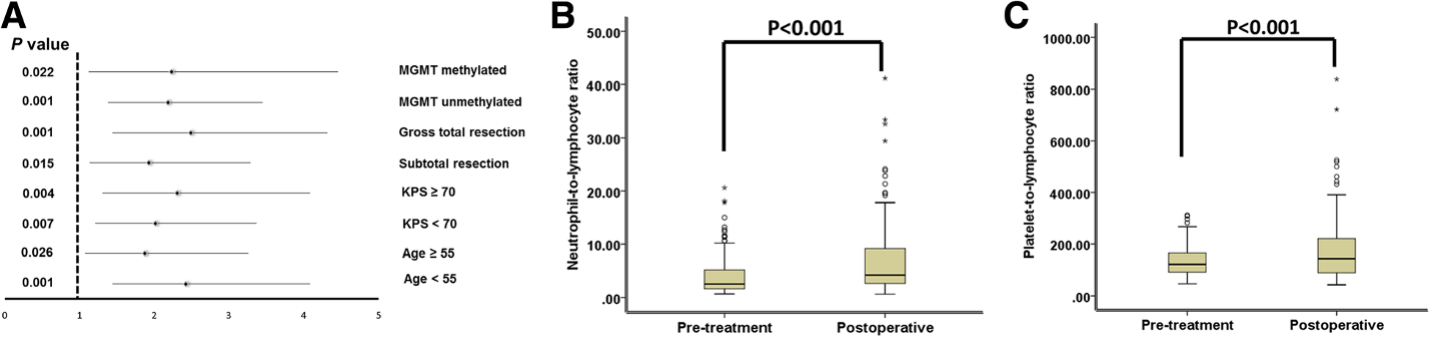
Correlation between pre-treatment NLR and other clinical factors in glioblastoma patients. **a** Stratified analysis of pre-treatment NLR level and overall mortality. Hazard ratios and 95 % confidence intervals in various strata are shown. **b** Relationship between pre-treatment NLR and postoperative NLR. **c** Relationship between pre-treatment PLR and postoperative PLR. NLR and PLR were assessed as continuous variables

Multivariate analysis showed that KPS and MGMT promoter methylation were also independently associated with OS in glioblastoma (Table 2). In keeping with prior literature and prognostic nomograms [27], variables including age (≤60 vs. >60) and KPS (≤70 vs. 80–100) were also analyzed as categorical variables. As shown in Tables 1 and 3, similar results were obtained. We further examined the influence of pre-treatment NLR on OS across strata of other potential predictors, including age, KPS, degree of resection, and MGMT promoter methylation status. Pre-treatment NLR was an independent prognostic factor in all the subgroups (Fig. 2a).

**Table 3:** Univariate and multivariate analyses of different prognostic parameters for overall survival of 152 glioblastoma patients

Variable	Univariate			Multivariate		
	*P*	HR	95 % CI	*P*	HR	95 % CI
Age (≤60 vs. >60)	0.025	1.603	1.060–2.424	0.478	1.168	0.755–1.806
KPS (≤70 vs. 80–100)	0.032	0.670	0.465–0.966	0.045	0.794	0.633–0.995
Resection (biopsy vs. STR vs. GTR)	0.047	0.800	0.641–0.997	0.110	0.738	0.509–1.071
MGMT promoter (unmethylated vs. methylated)	0.011	0.605	0.411–0.890	0.041	0.660	0.443–0.983
Pretreatment PLR (<135 vs. >135)	0.039	1.463	1.020–2.097	0.918	1.023	0.668–1.565
Pretreatment NLR (<4 vs. ≥4)	<0.001	2.139	1.464–3.125	0.002	2.068	1.304–3.277

*GTR* gross total resection, *KPS* Karnofsky Performance Scores, *MGMT* O(6)-methylguanine-DNA-methyltransferase, *NLR* Neutrophil to Lymphocyte Ratio, *PLR* Platelet to Lymphocyte Ratio, *STR* subtotal resection

However, postoperative NLR was not associated with patient outcome in glioblastoma (Table 2). After surgery, NLR increased significantly (mean ± SD: pre-treatment 4.1 ± 3.8 *vs.* postoperative 7.0 ± 6.7, *P* < 0.001; Fig. 2b) because of the effect of treatment, and so did PLR (pre-treatment 135.0 ± 57.1 *vs.* postoperative 177.7 ± 123.9, *P* < 0.001; Fig. 2c), which may affect their prognostic value. Moreover, consistent with a previous study [12], pre-treatment neutrophil count, lymphocyte count, and platelet count were not independently correlated with patient survival (Table 2).

### Pre-treatment NLR is superior to PLR as a prognostic factor in glioblastoma

**Fig. 3:**
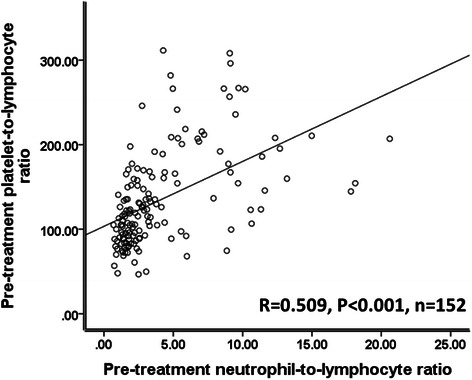
Pre-treatment NLR levels were significantly correlated with pre-treatment PLR levels. NLR and PLR were assessed as continuous variables

In the present study, we observed a significant correlation between the two systemic inflammatory markers, pre-treatment NLR and PLR (*R* = 0.509, *P* < 0.001; Table 1 and Fig. 3). In univariate analysis, both NLR and PLR (univariate hazard ratio = 1.004, 95 % confidence interval 1.001–1.007, *P* = 0.013, Tables 2 and 3) were associated with patient survival. However, in multivariate analysis, the prognostic significance of PLR was markedly diminished (Tables 2 and 3).

### Pre-treatment NLR and immune cell infiltration

**Fig. 4:**
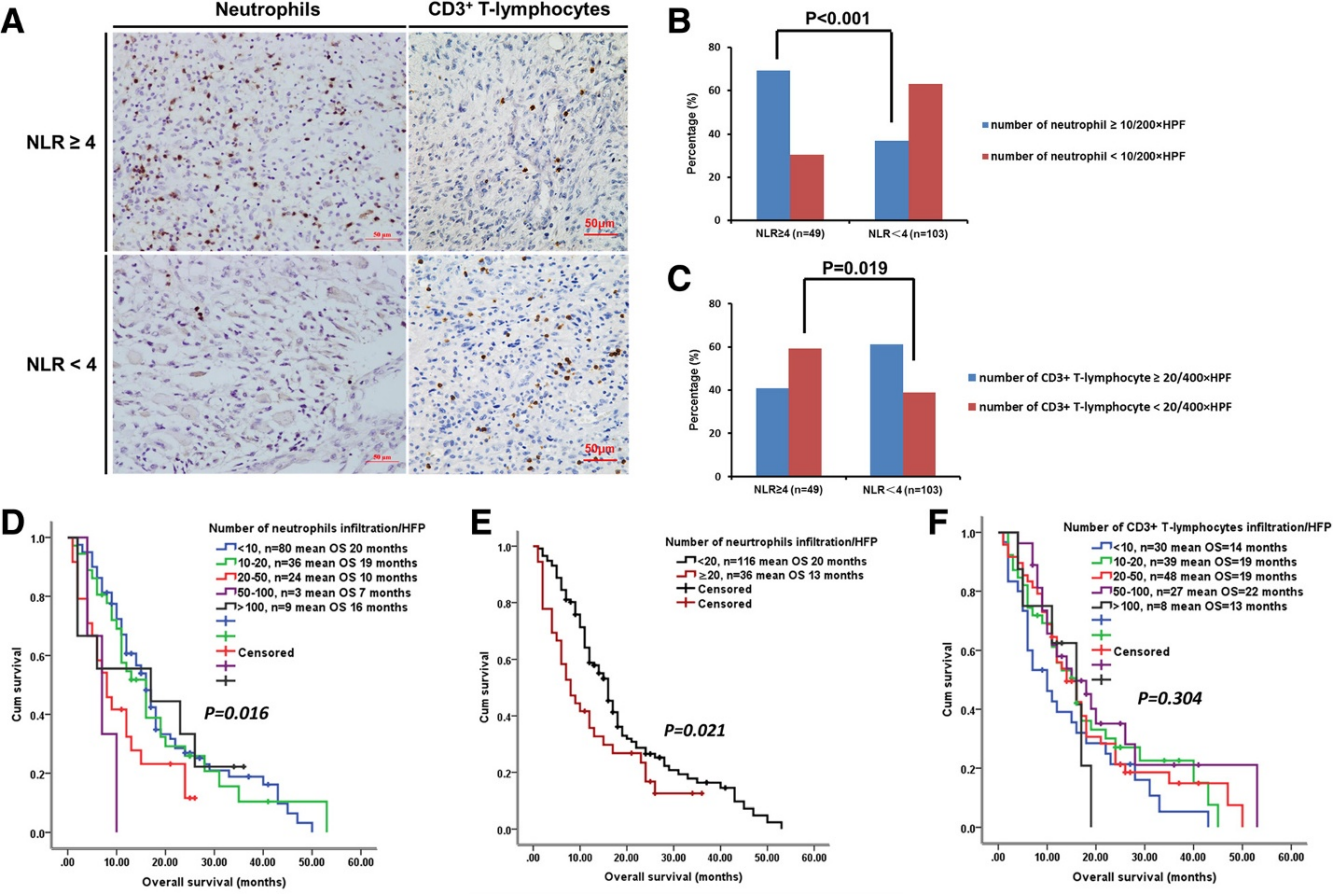
Correlation between pre-treatment NLR and immune cell infiltration in glioblastomas. **a** Representative immunohistochemical images of neutrophil infiltration and CD3^+^ T-cell infiltration in glioblastoma patients with pre-treatment NLR ≥4 and NLR <4. **b**, **c** Pre-treatment NLR ≥4 (versus NLR <4) significantly correlated with high neutrophil infiltration (**b**) and low CD3^+^ T-cell infiltration (**c**). High neutrophil infiltration (number of neutrophils ≥10/200 × HPF) and low CD3^+^ T-cell infiltration (number of CD3^+^ T-cells <20/400 × HPF) were more frequent in patients with pre-treatment NLR ≥4 than in those with pre-treatment NLR < 4 (69.4 vs. 36.9 %, *P* < 0.001 and 59.2 vs. 38.8 %, *P* = 0.019, respectively). **d**-**f** Kaplan-Meier plots of overall survival, stratified by different levels of neutrophil infiltration (D and E) or CD3^+^ T-cell infiltration (**f**). Scale bars, 50 μm

Tissue microarrays were used to assess neutrophil and T-cell infiltration using immunohistochemical staining in 152 glioblastomas. To evaluate the association between pre-treatment NLR and immune cell infiltration, we semi-quantified the infiltrating neutrophils and CD3^+^ T-cells. The level of neutrophil infiltration was significantly positively correlated with pre-treatment NLR level (NLR < 4 vs. NLR ≥ 4; *P* < 0.001), whereas CD3^+^ T-cell infiltration level was negatively correlated with pre-treatment NLR level (*P* = 0.006; Table 4). According to previous data and our results, a number of neutrophils ≥10/200 × HPF [8, 9] and a number of CD3^+^ T-cells ≥20/400 × HPF [7, 11] were used as cutoff points to define a high infiltration group and a low infiltration group. High neutrophil infiltration and low CD3^+^ T-cell infiltration were more frequent in patients with pre-treatment NLR ≥4 than in those with pre-treatment NLR < 4 (69.4 *vs.* 36.9 %, *P* < 0.001 and 59.2 *vs.* 38.8 %, *P* = 0.019, respectively (Fig. 4a-c).

**Table 4:** Correlation between neutrophils and CD3^+^ T-cells infiltration and pre-treatment NLR in 152 Glioblastomas

	Neutrophils infiltration (200 × HPF)			CD3^+^ T-cells infiltration (400 × HPF)		
	NLR ≥ 4 (*n* = 49)	NLR < 4 (*n* = 103)	*P*	NLR ≥ 4 (*n* = 49)	NLR < 4 (*n* = 103)	*P*
0 (<10)	15 (30.6 %)	65 (63.1 %)	<0.001	18 (36.7 %)	12 (11.7 %)	0.006
1 (10–20)	8 (16.3 %)	28 (27.2 %)		11 (22.5 %)	28 (27.2 %)	
2 (20–50)	17 (34.7 %)	7 (6.8 %)		11 (22.5 %)	37 (35.9 %)	
3 (50–100)	2 (4.1 %)	1 (1 %)		8 (16.3 %)	19 (18.4 %)	
4 (>100)	7 (14.3 %)	2 (1.9 %)		1 (2.0 %)	7 (6.8 %)

Kaplan-Meier analysis showed that increased neutrophil infiltration was correlated with shorter survival (log-rank, *P* = 0.016) (Fig. 4d-e). However, no association between CD3^+^ T-cell infiltration and OS was observed (log-rank *P* = 0.304) (Fig. 4f), which could be attributed to the complexity of its components.

## Discussion

The identification of prognostic factors is clinically significant for glioblastoma patients and can guide clinical treatment and studies [28]. Previous studies show a strong linkage between inflammation and cancer [29, 30]. Meanwhile, inflammatory markers have been associated with patient prognosis in glioblastoma [5, 7]. As a marker of systemic inflammation, pre-treatment NLR has been recognized as a prognostic factor in glioblastomas [12, 13]. However, this prognostic effect should be re-evaluated in the era of standard therapy [2], when molecular markers such as MGMT promoter methylation are taken into consideration [28, 31]. In the present study, NLR levels did not correlate with MGMT promoter methylation status, and the prognostic role of NLR was not significantly modified by MGMT promoter methylation status, suggesting that these two prognostic factors may influence clinical outcome via different pathways and mechanisms (Tables 1, 2 and 3).

Although pre-treatment NLR was an independent predictor of clinical outcome in glioblastoma patients, postoperative NLR had no prognostic value. Postoperatively, the NRL as well as the PLR level increased significantly (Fig. 2b-c), indicating that the stress of surgery had an impact on systemic inflammation. Thus, postoperative NLR cannot reflect the baseline impact of systemic inflammation on clinical outcome in glioblastoma patients. Moreover, some diseases, such as cardiovascular diseases and infection, or drug treatments might affect neutrophil and lymphocyte counts; therefore, the ratio of these two parameters might be changed [32]. To minimize potential confounders, patients with such medical history were excluded from this study. In addition, the NLR was measured before any treatment, including steroids. Therefore, our results may reflect the influence of basic systemic inflammation, possibly induced by the tumor, on the prognosis. Consistent with a previous study, we established a positive correlation between the two markers of systemic inflammatory responses, NLR and PLR [15]. Nevertheless, the prognostic value of NLR and PLR may vary among different cancers. In esophageal cancer, PLR is a better prognostic factor than NLR [15], whereas in endometrial cancer, NLR is a better prognostic factor [33]. We showed that pre-treatment NLR was superior to PLR as a prognostic factor in patients with glioblastoma. However, the underlying mechanism needs further research.

Definitive reasons for the association between elevated NLR and poor survival in cancer patients have not been identified to date. In the present study, we showed that high pre-treatment NLR (≥4 vs. < 4) was significantly associated with high neutrophil infiltration and low CD3^+^ T-cell infiltration into glioblastomas. Previous data show that neutrophil infiltration plays an important role in stimulating tumor growth, angiogenesis and metastasis [34–36]. In gliomas, there is a positive correlation between tumor grade and the extent of neutrophil infiltration [9]. Liang et al. reported that increased recruitment of neutrophils promotes glioma progression and treatment resistance [8]. Blocking neutrophil infiltration has been suggested for the treatment of glioblastoma [37]. Consistently, we found a correlation of increased neutrophil infiltration with shorter survival in glioblastoma patients (Fig. 4d-e). Furthermore, neutrophils may suppress the immune function [38] by inhibiting the cytolytic activity of CD8^+^ T-cells and natural killer cells [39, 40], and by enhancing the suppressive activities of CD4^+^ suppressor T cells [41]. T-lymphocytes play an important role in host defenses against tumors, as they inhibit the proliferation and invasion of tumor cells via the induction of cytotoxic cell death and cytokine production [30, 42]. Moreover, Kmiecik et al. reported an association between elevated CD3^+^ T-cell infiltration and prolonged survival in glioblastoma patients [11]. We found a negative correlation between pre-treatment NLR level and the number of infiltrating CD3^+^ T-cells; however, CD3^+^ T-cell infiltration was not associated with patient outcome. The complexity of CD3^+^ T-cell subpopulations may affect its prognostic significance. While tumor-infiltrating effector T cells (cytotoxic and helper) may correlate with a better survival, the association between tumor-infiltrating regulatory T cells and patient outcome remains unclear in glioblastoma [5, 7, 43, 44]. Our results indicate that, for glioblastoma patients, there could be a correlation and interaction between systemic and local inflammation, which may influence clinical outcome. In future studies, analysis of T-cell subsets would enable us to better understand this underlying relationship, and the molecular mechanism also needs to be explored.

The present study had several limitations. Firstly, the retrospective design of the study may lead to bias. Secondly, although a standard primary treatment regimen was applied, the post-progression salvage treatments were heterogeneous, which may have affected the survival analysis. Moreover, other unknown physiological and pathophysiological factors potentially affecting NLR may have influenced our analysis.

## Conclusion

We showed that pre-treatment NLR was superior to PLR as a predictor of clinical outcome in patients with glioblastoma. NLR is of prognostic significance independent of MGMT status. Our results demonstrate a correlation between elevated peripheral blood NLR levels and increased tumor neutrophil infiltration/decreased CD3^+^T-cell infiltration. The association of NLR in the peripheral blood with immune cell infiltration in the tumor microenvironment provides insight into the mechanism by which NLR can predict prognosis.

## Abbreviations

BMI: Body mass index
HPF: High-power fields
HR: Hazard ratio
KPS: Karnofsky performance status
MGMT: O-methylguanine-DNA methyltransferase
NLR: Neutrophil-to-lymphocyte ratio
OS: Overall survival
PLR: Platelet-to-lymphocyte ratio
TMZ: Temozolomide

## References

[1] Louis DN, Ohgaki H, Wiestler OD, Cavenee WK, Burger PC, Jouvet A (2007). The 2007 WHO classification of tumours of the central nervous system. Acta Neuropathol.

[2] Stupp R, Mason WP, van den Bent MJ, Weller M, Fisher B, Taphoorn MJ (2005). Radiotherapy plus concomitant and adjuvant temozolomide for glioblastoma. N Engl J Med.

[3] Aum DJ, Kim DH, Beaumont TL, Leuthardt EC, Dunn GP, Kim AH (2014). Molecular and cellular heterogeneity: the hallmark of glioblastoma. Neurosurg Focus.

[4] Han S, Meng L, Han S, Wang Y, Wu A (2014). Plasma IGFBP-2 levels after postoperative combined radiotherapy and chemotherapy predict prognosis in elderly glioblastoma patients. PLoS One.

[5] Han S, Zhang C, Li Q, Dong J, Liu Y, Huang Y (2014). Tumour-infiltrating CD4(+) and CD8(+) lymphocytes as predictors of clinical outcome in glioma. Br J Cancer.

[6] Rutledge WC, Kong J, Gao J, Gutman DA, Cooper LA, Appin C (2013). Tumor-infiltrating lymphocytes in glioblastoma are associated with specific genomic alterations and related to transcriptional class. Clin Cancer Res.

[7] Lohr J, Ratliff T, Huppertz A, Ge Y, Dictus C, Ahmadi R (2011). Effector T-cell infiltration positively impacts survival of glioblastoma patients and is impaired by tumor-derived TGF-beta. Clin Cancer Res.

[8] Liang J, Piao Y, Holmes L, Fuller GN, Henry V, Tiao N (2014). Neutrophils promote the malignant glioma phenotype through S100A4. Clin Cancer Res.

[9] Fossati G, Ricevuti G, Edwards SW, Walker C, Dalton A, Rossi ML (1999). Neutrophil infiltration into human gliomas. Acta Neuropathol.

[10] Iwatsuki K, Kumara E, Yoshimine T, Nakagawa H, Sato M, Hayakawa T (2000). Elastase expression by infiltrating neutrophils in gliomas. Neurol Res.

[11] Kmiecik J, Poli A, Brons NH, Waha A, Eide GE, Enger PØ (2013). Elevated CD3+ and CD8+ tumor-infiltrating immune cells correlate with prolonged survival in glioblastoma patients despite integrated immunosuppressive mechanisms in the tumor microenvironment and at the systemic level. J Neuroimmunol.

[12] Bambury RM, Teo MY, Power DG, Yusuf A, Murray S, Battley JE (2013). The association of pre-treatment neutrophil to lymphocyte ratio with overall survival in patients with glioblastoma multiforme. J Neurooncol.

[13] McNamara MG, Lwin Z, Jiang H, Templeton AJ, Zadeh G, Bernstein M (2014). Factors impacting survival following second surgery in patients with glioblastoma in the temozolomide treatment era, incorporating neutrophil/lymphocyte ratio and time to first progression. J Neurooncol.

[14] Szkandera J, Absenger G, Liegl-Atzwanger B, Pichler M, Stotz M, Samonigg H (2013). Elevated preoperative neutrophil/lymphocyte ratio is associated with poor prognosis in soft-tissue sarcoma patients. Br J Cancer.

[15] Feng JF, Huang Y, Chen QX (2014). Preoperative platelet lymphocyte ratio (PLR) is superior to neutrophil lymphocyte ratio (NLR) as a predictive factor in patients with esophageal squamous cell carcinoma. World J Surg Oncol.

[16] Lee YY, Choi CH, Kim HJ, Kim TJ, Lee JW, Lee JH (2012). Pretreatment neutrophil:lymphocyte ratio as a prognostic factor in cervical carcinoma. Anticancer Res.

[17] Boyraz I, Koc B, Boyaci A, Tutoglu A, Sarman H, Ozkan H (2014). Ratio of neutrophil/lymphocyte and platelet/lymphocyte in patient with ankylosing spondylitis that are treating with anti-TNF. Int J Clin Exp Med.

[18] Buyukkaya E, Karakas MF, Karakas E, Akçay AB, Tanboga IH, Kurt M (2014). Correlation of neutrophil to lymphocyte ratio with the presence and severity of metabolic syndrome. Clin Appl Thromb Hemost.

[19] Yasar Z, Buyuksirin M, Ucsular FD, Kargı A, Erdem F, Talay F (2015). Is an elevated neutrophil-to-lymphocyte ratio a predictor of metabolic syndrome in patients with chronic obstructive pulmonary disease. Eur Rev Med Pharmacol Sci.

[20] Belen E, Sungur A, Sungur MA, Erdogan G. Increased Neutrophil to Lymphocyte Ratio in Patients With Resistant Hypertension.LID. J Clin Hypertens (Greenwich). 2015. doi: 10.1111/jch.12533 10.1111/jch.12533 PMC8031939 25807989

[21] Balta S, Demirkol S, Ozturk C (2014). The neutrophil lymphocyte ratio in patients with glioblastoma multiforme. J Neurooncol.

[22] Liu X, Zhang Q, Wu H, Du H, Liu L, Shi H, et al. Blood Neutrophil to Lymphocyte Ratio as a Predictor of Hypertension.LID. Am J Hypertens. 2015. Epub ahead of print 10.1093/ajh/hpv034 25824450

[23] Han S, Huang Y, Wang Z, Li Z, Qin X, Wu A (2014). Increased rate of positive penicillin skin tests among patients with glioma: insights into the association between allergies and glioma risk. J Neurosurg.

[24] Han S, Xia J, Qin X, Han S, Wu A (2013). Phosphorylated SATB1 is associated with the progression and prognosis of glioma. Cell Death Dis.

[25] Esteller M, Hamilton SR, Burger PC, Baylin SB, Herman JG (1999). Inactivation of the DNA repair gene O6-methylguanine-DNA methyltransferase by promoter hypermethylation is a common event in primary human neoplasia. Cancer Res.

[26] Lin Y, Jiang T, Zhou K, Xu L, Chen B, Li G (2009). Plasma IGFBP-2 levels predict clinical outcomes of patients with high-grade gliomas. Neuro Oncol.

[27] Gorlia T, van den Bent MJ, Hegi ME, Mirimanoff RO, Weller M, Cairncross JG (2008). Nomograms for predicting survival of patients with newly diagnosed glioblastoma: prognostic factor analysis of EORTC and NCIC trial 26981-22981/CE.3. Lancet Oncol.

[28] Stupp R, Brada M, van den Bent MJ, Tonn JC, Pentheroudakis G (2014). High-grade glioma: ESMO Clinical Practice Guidelines for diagnosis, treatment and follow-up. Ann Oncol.

[29] Colotta F, Allavena P, Sica A, Garlanda C, Mantovani A (2009). Cancer-related inflammation, the seventh hallmark of cancer: links to genetic instability. Carcinogenesis.

[30] Mantovani A, Allavena P, Sica A, Balkwill F (2008). Cancer-related inflammation. Nature.

[31] Wick W, Weller M, van den Bent M, Sanson M, Weiler M, von Deimling A (2014). MGMT testing--the challenges for biomarker-based glioma treatment. Nat Rev Neurol.

[32] Bhat T, Teli S, Rijal J, Bhat H, Raza M, Khoueiry G (2013). Neutrophil to lymphocyte ratio and cardiovascular diseases: a review. Expert Rev Cardiovasc Ther.

[33] Haruma T, Nakamura K, Nishida T, Ogawa C, Kusumoto T, Seki N (2015). Pre-treatment neutrophil to lymphocyte ratio is a predictor of prognosis in endometrial cancer. Anticancer Res.

[34] Jablonska J, Leschner S, Westphal K, Lienenklaus S, Weiss S (2010). Neutrophils responsive to endogenous IFN-beta regulate tumor angiogenesis and growth in a mouse tumor model. J Clin Invest.

[35] Nozawa H, Chiu C, Hanahan D (2006). Infiltrating neutrophils mediate the initial angiogenic switch in a mouse model of multistage carcinogenesis. Proc Natl Acad Sci U S A.

[36] Van den Eynden GG, Majeed AW, Illemann M, Vermeulen PB, Bird NC, Høyer-Hansen G (2013). The multifaceted role of the microenvironment in liver metastasis: biology and clinical implications. Cancer Res.

[37] Kast RE, Scheuerle A, Wirtz CR, Karpel-Massler G, Halatsch ME (2011). The rationale of targeting neutrophils with dapsone during glioblastoma treatment. Anticancer Agents Med Chem.

[38] el-Hag A, Clark RA (1987). Immunosuppression by activated human neutrophils. Dependence on the myeloperoxidase system. J Immunol.

[39] Petrie HT, Klassen LW, Kay HD (1985). Inhibition of human cytotoxic T lymphocyte activity in vitro by autologous peripheral blood granulocytes. J Immunol.

[40] Shau HY, Kim A (1988). Suppression of lymphokine-activated killer induction by neutrophils. J Immunol.

[41] Hirohata S, Yanagida T, Yoshino Y, Miyashita H (1995). Polymorphonuclear neutrophils enhance suppressive activities of anti-CD3-induced CD4+ suppressor T cells. Cell Immunol.

[42] Coussens LM, Werb Z (2002). Inflammation and cancer. Nature.

[43] Kim YH, Jung TY, Jung S, Jang WY, Moon KS, Kim IY (2012). Tumour-infiltrating T-cell subpopulations in glioblastomas. Br J Neurosurg.

[44] Yue Q, Zhang X, Ye HX, Wang Y, Du ZG, Yao Y (2014). The prognostic value of Foxp3+ tumor-infiltrating lymphocytes in patients with glioblastoma. J Neurooncol.

